# A Survey of Neurosurgery Residency Program Coordinators: Their Roles, Responsibilities, and Perceived Value

**DOI:** 10.7759/cureus.4457

**Published:** 2019-04-14

**Authors:** Brenden Ronna, Jian Guan, Michael Karsy, Julie Service, Amy Ekins, Randy Jensen

**Affiliations:** 1 Department of Neurosurgery, University of Utah, Salt Lake City, USA; 2 Department of Neurosurgery, University of Utah , Salt Lake City, USA

**Keywords:** residency programs, accreditation council for graduate medical education, program coordinator, program administrator, program director, fellowship program, neurological surgery, neurological surgery residency

## Abstract

Objective

Residency program coordinators play an important role behind the scenes, in the function of residency and fellowship programs. In addition, coordinators have significantly heterogeneous job roles among institutions. The aim of this study was to evaluate the training, responsibilities, and contribution of residency program coordinators within the field of neurosurgery.

Methods

A 24-question survey was submitted to 133 program coordinators, and 78 responses (59% response rate) were received.

Results

The survey results showed that >80% of coordinators have been in their current position for ≥3 years. Coordinators identified at least 24 unique departmental responsibilities with an average of 85% of the time devoted to residency program management. Among coordinators, 82% reported no formal training, with 60% and 55% reporting inadequate training from their department and institution, respectively. Interestingly, 84% completely or partially agreed that their work is valued by residents, 91% by the program director(s), 78% by the department chair, 62% by other faculty, and 56% by other departmental staff. Lastly, 50% of coordinators reported that their department has not been receptive to receiving feedback on how to improve the roles of the position, with 80% reporting no career advancement track.

Conclusion

Residency program coordinators reported a wide range of experience and responsibilities within their respective departments. The majority reported limited training for their current position, and a significant number reported not feeling valued by members of their department, suggesting two areas for improvement. As coordinators continue to play a larger role in the management and accreditation of their departments, strategies to optimize their role may be important.

## Introduction

Neurosurgery residency program coordinators play an important role in the organization and function of residency and fellowship programs. In addition to managing the daily operation of these programs, coordinators must manage Accreditation Council for Graduate Medical Education (ACGME) requirements for program accreditation in direct coordination with the program director(s), department chair, and other department staff [1-3]. First formalized in 2008, the Society of Neurosurgical Surgeons Coordinator’s Group grew out of the increasingly formalized ACGME requirements for neurosurgery departments. Continued expansion of ACGME since its founding in 1981 has led to the increased complexity of accreditation requirements, site visits, and annual reviews.

Currently, the ACGME Next Accreditation System, formed in 2015, governs the process of residency and fellowship program management. The demand placed on residency program coordinators has increased over the last decade as the ACGME requirements and other regulatory governance in neurosurgery programs have become more complex [4-5]. These changes have resulted in an expansion of the program coordinator’s role from a secretarial responsibility to a managerial position involved in budgeting, departmental operations, human resource (i.e., for residents and fellows), management, and regulatory affairs [4-6]. Furthermore, as neurosurgery residency programs have grown in size and complexity (to 115 ACGME-accredited programs in the U.S. with a total of 1479 residents [7]), the residency program directors and coordinators have greater responsibility to facilitate training and administrative tasks.

Currently, there are several established Graduate Medical Education (GME) and ACGME requirements and guidelines for program coordinators, but except for the requirement that there must be a program coordinator with at least 50% of his or her time dedicated to the residency program, these are not consistent across specialties [1,3,8]. The only specified ACGME requirement for neurosurgical program coordinators states, “There should be a full-time designated program coordinator with financial support from the sponsoring institution” [9]. Thus, the role of the program coordinator is specified by individual institutions, leading to heterogeneity in program coordinator training and duties.

Despite the complex responsibilities of program coordinators, surveys among coordinators in other medical fields have shown that training for the position from institutions and departments is often not sufficient [1-2]. The aim of this survey was to evaluate the experience, training, responsibilities, and contribution of neurosurgery residency program coordinators.

## Materials and methods

An anonymous 24-question survey was prepared for distribution to residency coordinators throughout the U.S. This survey was sent to 133 program coordinators working at ACGME-accredited neurosurgical residency programs.

The survey included nine questions assessing the demographics of residency program coordinators, two questions about current roles and responsibilities, 12 questions surrounding the value of work and contribution, and one final question allowing coordinators to provide any information about their job they would add in addition to the survey (Table 1). Job responsibilities were assessed by asking coordinators to select which of 24 common roles and responsibilities they perform as part of their job, along with one blank answer space allowing participants to add any roles that were not listed. Value and contribution were surveyed through questions assessing the coordinators’ subjective views on how the institution and department view their work as well as how individuals within the department value their contributions.

**Table 1:** Survey questionnaire

How many years have you worked in your current position?
Did you have formal training for your job as a program coordinator/administrator?
Are you certified in Training Administrators of Graduate Medical Education (TAGME)?
Do you feel that your training for the program coordinator/administrator position was adequate from your DEPARTMENT?
Do you feel that your training for the program coordinator/administrator position was adequate from your INSTITUTION?
What is the total number of residency and fellowship programs you currently manage? (Count the residency as 1 program)
How many hours per week do you work?
Do you handle more than 1 department?
What are your current duties (check all that apply)?
What other duties are you involved in?
What percentage of your time is your role as a residency/fellowship coordinator?
Do you feel valued by your Institution? Department? Chairperson? Program director? Other faculty? Fellows? Residents? Other staff?
Is there a career path for advancement at your department?
Is there an institutional committee for organizing program coordinators/administrators?
Has the INSTITUTION been responsive to feedback for improving the role and jobs of program coordinators/administrators?
Has the DEPARTMENT been responsive to feedback for improving the role and jobs of program coordinators/administrators?
Is there additional information about your job/role that you wish others knew about or any other comments about this survey? Free text

Survey responses were received and analyzed using REDCap (Tennessee, US) and Microsoft Excel (Microsoft Corp., Washington, US). Continuous and discrete variables were analyzed using the T-test and Chi-squared test, respectively. The linear correlation of continuous variables was performed. All statistical analyses were completed using SPSS v22.0 (IBM Corp, Armonk, NY, US) with p<0.05 considered statistically significant.

## Results

Residency program coordinator demographics

A total of 78 coordinators completed and returned the survey (59% response rate) (Table 2). More than 50% of coordinators reported working in their current job for more than five years. Most (79.5%) reported managing ≤3 residency/fellowship programs, and the management of residency/fellowship programs constituted 85% of their job time on average. A majority (82.1%) of coordinators stated that they received no formal training for their current job, and only 11 (14.1%) reported that they are currently certified as Training Administrators of Graduate Medical Education (TAGME). Interestingly, 47 (60.3%) coordinators noted that their training was not adequate from their neurosurgery department and 43 (55.1%) coordinators reported receiving inadequate training from their institution.

**Table 2:** Neurosurgery program coordinator demographics

Years worked in current position	<1	1-2	3–5	5–10	10–15	>15
	5 (6.4%)	10 (12.8%)	19 (24.4%)	19 (24.4%)	10 (12.8%)	15 (19.2%)
How many residency and fellowships programs do you currently manage?	1	2	3	4	5	>5
	28 (35.9%)	17 (21.8%)	17 (21.8%)	7 (9.0%)	5 (6.4%)	4 (5.1%)
How many hours per week do you work?	20	20–30	30–40	40–50	>50	
	0 (0.0%)	0 (0.0%)	15 (19.2%)	56 (71.8%)	7 (9.0%)	
What percentage of your time is your role as a residency/ fellowship coordinator?						84.7±19.2
Did you have formal training for your job as a program coordinator/administrator?						14 (17.9%)
Are you certified in Training Administrators of Graduate Medical Education?						11 (14.1%)
Do you feel that your training for the program coordinator/administrator position was adequate from your department?						31 (39.7%)
Do you feel that your training for the program coordinator/administrator position was adequate from your institution?						35 (44.9%)
Do you handle more than one department?						10 (12.8%)

Roles and responsibilities

More than 85% of the responding residency program coordinators reported that their job duties included coordinating with the Electronic Residency Application Service (ERAS), Graduate Medical Education (GME), and National Resident Matching Program (NRMP) offices; participating in interview scheduling; completing paperwork for ACGME accreditation; providing residency orientation; completing rotation scheduling; and attending resident-program director meetings (Table 3). Fewer than 50% of coordinators reported that human resources or working as a coordinator for a clinician or faculty member was part of their duties.

**Table 3:** Current program coordinator duties ACGME: Accreditation Council for Graduate Medical Education; CAST: Committee on Advanced Subspecialty Training; FRIEDA: Fellowship and Residency Electronic Interactive Database Access; GME: Graduate Medical Education; ERAS: Electronic Residency Application Service; NRMP: National Resident Matching Program

Duty	Number performing this duty	Percentage reporting this duty
Coordination with GME office	77	98.7
Paperwork for residency ACGME accreditation	77	98.7
Resident evaluations	77	98.7
Interview scheduling	76	97.4
Resident permanent files maintenance	76	97.4
Resident–program director meetings	75	96.2
Coordination with ERAS office	73	93.6
Program updates and announcements	73	93.6
Residency committee meetings	73	93.6
Resident in-service exam planning	73	93.6
Faculty evaluations	70	89.7
Coordination with NRMP office	69	88.5
Paperwork for other credentialing documentation	69	88.5
Resident orientation	68	87.2
Resident case logs maintenance	67	85.9
Resident rotation scheduling	67	85.9
Management of book/travel funds	66	84.6
Coordination with FRIEDA office	60	76.9
Other	53	67.9
Program website maintenance	53	67.9
Visa/immigration issues	51	65.4
Fellowship CAST approval paperwork	48	61.5
Call schedule management	41	52.6
Human resources	34	43.6
Administrator for clinician or faculty	33	42.3

Self-reported responsibilities listed under “other duties” included Continuing Medical Education (CME) coordination, scheduling visiting lecturers or professors, organization of medical student away and home rotations and shadowing, and other administrative tasks to include departmental budgeting, grants and awards, and departmental operations management.

Value and contribution

We assessed the self-perceived valuation indicated by program coordinators (Table 4). Fewer than <20% of coordinators reported a career advancement track through their department. With regard to value and contribution, 63% of coordinators completely or partially agreed that their work is valued by their institution, 75% by their department, 78% by the department chair, 91% by the program director(s), 62% by other faculty, 61% by fellows, 84% by residents, and 56% by other staff. A total of 48.7% of coordinators reported that the institution has been responsive to feedback for improving their role or job and 50.0% report that their department has been responsive.

**Table 4:** Institutional validation of program coordinators

Question (number responding)	Completely Disagree	Somewhat Disagree	Neutral	Somewhat Agree	Completely Agree
Do you feel that you are valued by your institution? N=76	8 (10.5%)	7 (9.2%)	13 (17.1%)	23 (30.3%)	25 (32.9%)
Do you feel that you are valued by your department? N=77	1 (1.3%)	8 (10.4%)	8 (10.4%)	32 (41.6%)	28 (32.9%)
Do you feel that you are valued by your chairperson? N=76	2 (2.6%)	6 (7.9%)	9 (11.8%)	22 (28.9%)	37 (48.7%)
Do you feel that you are valued by your program director? N=77	0 (0.0%)	5 (6.5%)	2 (2.6%)	18 (23.4%)	52 (67.5%)
Do you feel that you are valued by other faculty? N=77	2 (2.6%)	7 (9.1%)	20 (26.0%)	28 (36.4%)	20 (26.0%)
Do you feel that you are valued by fellows? N=67	0 (0.0%)	4 (6.0%)	22 (32.8%)	17 (25.4%)	24 (35.8%)
Do you feel that you are valued by residents? N=77	0 (0.0%)	3 (3.9%)	9 (11.7%)	20 (26.0%)	45 (58.4%)
Do you feel that you are valued by other staff? N=75	4 (5.3%)	9 (12.0%)	20 (26.7%)	25 (33.3%)	17 (22.7%)
				Yes	No
Is there a career path for advancement at your department?				15 (19.2%)	63 (80.8%)
Is there an institutional committee for organizing program coordinators/administrators?				60 (76.9%)	18 (23.1%)
Has the institution been responsive to feedback for improving the role and jobs of program coordinators/administrators?				38 (48.7%)	40 (51.3%)
Has the department been responsive to feedback for improving the role and jobs of program coordinators/administrators?				39 (50.0%)	39 (50.0%)

The self-reported valuation of coordinators was correlated among staff categories (Table 5). That is, coordinators who somewhat or completely agreed that they felt valued by their institution or department reported the same valuation by the department chair, program director(s), and staff. Next, we evaluated the responses of coordinators that felt somewhat or completely undervalued was performed (Table 6). Coordinators who felt undervalued by their institution were less likely to report adequate institutional training (20.0 vs. 50.0%, p=0.04), managed more programs (p=0.01), and reported poor institutional responsiveness to feedback (13.3 vs. 64.6%, p=0.001) compared with those who were valued. Program coordinators who felt undervalued by their departments described limited departmental training (11.1 vs. 45.0%, p=0.05) and limited responsiveness of departments to feedback (11.1 vs. 60.0%, p=0.006) as compared with individuals who felt valued. For coordinators who felt undervalued by their department chair, a lower level of departmental responsiveness to feedback was seen (12.5% vs. 54.2%, p=0.03). Coordinators who felt undervalued by their program director(s) had spent shorter lengths of time at their current positions (p=0.006) and reported less departmental training (0.0 vs. 43.3%, p=0.05). No distinguishing factors were seen for residency program coordinators who reported feeling undervalued by other faculty, fellows, residents, or other staff.

**Table 5:** Correlation of perceived coordinator value r: correlation coefficient, 2-tailed Spearman correlation

		Department	Chairperson	Program director	Other faculty	Fellows	Residents	Other staff
Institution	r	0.475	0.375	0.231	0.217	0.257	0.402	0.353
	P-value	0.0001	0.001	0.045	0.06	0.036	0	0.002
Department	r		0.598	0.413	0.298	0.39	0.303	0.443
	P-value		0.0001	0.0001	0.008	0.001	0.007	0.0001
Chairperson	r			0.378	0.476	0.411	0.476	0.576
	P-value			0.001	0.0001	0.001	0.0001	0.0001
Program director	r				0.478	0.341	0.42	0.273
	P-value				0.0001	0.005	0.0001	0.018
Other faculty	r					0.5	0.53	0.418
	P-value					0.0001	0.0001	0.0001
Fellows	r						0.638	0.491
	P-value						0.0001	0.0001
Residents	r							0.316
	P-value							0.006

**Table 6:** Factors impacting perceived valuation of program coordinators TAGME, Training Administrators of Graduate Medical Education

	Institution			Dept			Chairperson			Program director			Other faculty			Fellow			Residents			Other staff		
	Unvalued N=15	Valued N=48	P-value	Unvalued N=9	Valued N=60	P-value	Unvalued N=8	Valued N=59	P-value	Unvalued N=5	Valued N=48	P-value	Unvalued N=9	Valued N=48	P-value	Unvalued N=4	Valued N=41	P-value	Unvalued N=3	Valued N=65	P-value	Unvalued N=13	Valued N=42	P-value
Years in position			0.8			0.7			0.9			0.006			0.3			0.5			0.2			0.4
<1	1 (6.7%)	1 (2.1%)		1 (11.1%)	4 (6.7%)		0 (0.0%)	2 (3.4%)		1 (20.0%)	3 (4.3%)		0 (0.0%)	3 (6.2%)		1 (25.0%)	2 (4.9%)		1 (33.3%)	3 (4.6%)		1 (7.7%)	4 (9.5%)	
1-2	2 (13.3%)	6 (12.5%)		1 (11.1%)	8 (13.3%)		1 (12.5%)	7 (11.9%)		2 (40.0%)	8 (11.4%)		3 (33.3%)	6 (12.5%)		1 (25.0%)	5 (12.2%)		1 (33.3%)	7 (10.8%)		2 (15.4%)	6 (14.3%)	
3-5	3 (20.0%)	12 (25.0%)		2 (22.2%)	15 (25.0%)		1 (12.5%)	17 (28.8%)		0 (0.0%)	19 (27.1%)		1 (11.1%)	12 (25.0%)		0 (0.0%)	10 (24.4%)		0 (0.0%)	18 (27.7%)		4 (30.8%)	9 (21.4%)	
5-10	4 (26.7%)	11 (22.9%)		7 (77.8%)	15 (25.0%)		2 (25.0%)	13 (22.0%)		1 (20.0%)	17 (24.3%)		4 (44.4%)	12 (25.0%)		1 (25.0%)	11 (26.8%)		1 (33.3%)	14 (21.5%)		3 (23.1%)	9 (21.4%)	
10-15	1 (6.7%)	9 (18.8%)		0 (0.0%)	9 (15.0%)		2 (25.0%)	8 (13.6%)		0 (0.0%)	10 (14.3%)		1 (11.1%)	7 (14.6%)		1 (25.0%)	7 (17.1%)		0 (0.0%)	9 (13.8%)		2 (15.4%)	7 (16.7%)	
>15	4 (26.7%)	9 (18.8%)		3 (33.3%)	9 (15.0%)		2 (25.0%)	12 (20.3%)		1 (20.0%)	13 (18.6%)		0 (0.0%)	8 (16.7%)		0 (0.0%)	6 (14.6%)		0 (0.0%)	14 (21.5%)		1 (7.7%0	7 (16.7%)	
Formal training?	4 (26.7%0	8 (16.7%)	0.4	2 (22.2%)	10 (16.7%)	0.7	3 (37.5%)	10 (16.9%)	0.2	1 (20.0%)	13 (18.6%)	0.9	1 (11.1%)	8 (16.7%)	0.7	1 (25.0%)	7 (17.1%)	0.7	0 (0.0%)	12 (18.5%)	0.4	2 (15.4%0	10 (23.8%)	0.5
TAGME?	2 (13.3%)	9 (18.8%)	0.6	2 (22.2%)	9 (15.0%)	0.6	2 (25.0%)	9 (15.3%)	0.5	0 (0.0%)	11 (15.7%)	0.3	1 (11.1%)	8 (16.7%)	0.7	0 (0.0%)	5 (12.2%)	0.5	0 (0.0%)	10 (15.4%)	0.5	1 (7.7%)	6 (14.3%)	0.5
Adequate departmental training?	4 (26.7%)	24 (50.0%)	0.1	1 (11.1%)	27 (45.0%)	0.05	2 (25.0%)	26 (44.1%)	0.3	0 (0.0%)	31 (43.3%)	0.05	3 (33.3%)	23 (47.9%)	0.4	1 (25.0%)	18 (43.9%)	0.5	0 (0.0%)	28 (43.1%)	0.1	4 (30.8%)	20 (47.6%)	0.3
Adequate institutional training?	3 (20.0%)	24 (50.0%)	0.04	5 (55.6%)	26 (43.3%)	0.5	3 (37.5%)	27 (45.8%)	0.7	2 (40.0%)	31 (44.3%)	0.9	3 (33.3%)	24 (50.0%)	0.8	3 (75.0%)	20 (48.8%)	0.3	3 (100.0%)	29 (44.6%)	0.06	4 (30.8%)	24 (57.1%)	0.1
Number programs managed			0.01			0.7			0.3			0.5			0.2			0.1			0.2			0.5
1	5 (33.3%)	18 (37.5%)		5 (55.6%)	19 (31.7%)		4 (50.0%)	21 (35.6%)		3 (60.0%)	25 (35.7%)		2 (22.2%)	19 (39.6%)		1 (25.0%)	8 (19.5%)		1 (33.3%)	24 (36.9%)		4 (30.8%)	14 (33.3%)	
2	2 (13.3%)	10 (20.8%)		1 (11.1%)	13 (21.7%)		0 (0.0%)	12 (20.3%)		1 (20.0%)	15 (21.4%)		2 (22.2%)	11 (22.9%)		0 (0.0%)	9 (22.0%)		0 (0.0%)	13 (20.0%)		2 (15.4%)	9 (21.4%)	
3	3 (20.0%)	12 (25.0%)		1 (11.1%)	16 (26.7%)		1 (12.5%)	15 (25.4%)		0 (0.0%)	17 (24.3%)		1 (11.1%)	12 (25.0%)		0 (0.0%)	14 (34.1%)		0 (0.0%)	17 (26.2%)		3 (23.1%)	12 (28.6%)	
4	1 (6.7%)	5 (10.4%)		1 (11.1%)	5 (8.3%)		2 (25.0%)	4 (6.8%)		0 (0.0%)	6 (8.6%)		2 (22.2%)	2 (4.2%)		2 (50.0%)	3 (7.3%)		1 (33.3%)	4 (6.2%)		2 (15.4%)	2 (4.8%)	
5	4 (26.7%)	0 (0.0%)		1 (11.1%)	4 (6.7%)		1 (12.5%)	4 (6.8%)		1 (20.0%)	4 (5.7%)		2 (22.2%)	3 (6.2%)		1 (25.0%)	4 (9.8%)		1 (33.3%)	4 (6.2%)		2 (15.4%)	2 (4.8%)	
>5	0 (0.0%)	3 (6.2%)		0 (0.0%)	3 (5.0%)		0 (0.0%)	3 (5.1%)		0 (0.0%)	3 (4.3%)		0 (0.0%)	1 (2.1%)		0 (0.0%)	3 (7.3%)		0 (0.0%)	4 (6.2%)		0 (0.0%)	3 (7.1%)	
Hours per week			0.3			0.4			0.3			0.7			0.5			0.7			0.3			0.6
30-40	2 (13.3%)	10 (20.8%)		1 (11.1%)	12 (20.0%)		1 (12.5%)	12 (20.3%)		1 (20.0%)	14 (20.0%)		2 (22.2%)	12 (25.0%)		1 (25.0%)	8 (19.5%)		1 (33.3%)	12 (18.5%)		3 (23.1%)	8 (19.0%)	
40-50	10 (66.7%)	35 (72.9%)		6 (66.7%)	43 (71.7%)		5 (62.5%)	42 (71.2%)		3 (60.0%)	50 (71.4%)		5 (55.6%)	32 (66.7%)		2 (50.0%)	28 (68.3%)		1 (33.3%)	47 (72.3%)		8 (61.5%)	31 (73.8%)	
>50	3 (20.0%)	3 (6.2%)		2 (22.2%)	5 (8.3%)		2 (25.0%)	5 (8.5%)		1 (20.0%)	6 (8.6%)		2 (22.2%)	4 (8.3%)		1 (25.0%)	5 (12.2%)		1 (33.3%)	6 (9.2%)		2 (15.4%)	3 (7.1%)	
% of time program coordinator	92±12	84±20	0.06	71±28	87±18	0.1	77±25	87±18	0.2	80±15	85±19	0.6	91±13	83±21	0.2	86±18	84±19	0.1	81±18	86±19	0.7	87±21	85±18	0.7
Handle >1 dept?	3 (20.0%)	6 (12.5%)	0.5	1 (11.1%)	9 (15.0%)	0.8	0 (0.0%)	9 (15.3%)	0.2	1 (20.0%)	9 (12.9%)	0.7	1 (11.1%)	7 (14.6%)	0.8	0 (0.0%)	7 (17.1%)	0.4	0 (0.0%)	9 (13.8%)	0.5	1 (7.7%)	6 (14.3%)	0.5
Career advancement in dept?	1 (6.7%)	13 (27.1%)	0.1	1 (11.1%)	14 (23.3%)	0.4	1 (12.5%)	13 (22.0%)	0.4	1 (20.0%)	14 (20.0%)	1.0	1 (11.1%)	8 (16.7%)	0.7	1 (25.0%)	9 (22.0%)	0.9	1 (33.3%)	13 (20.0%)	0.6	1 (7.7%)	11 (26.2%)	0.2
Institutional organizing program?	10 (66.7%)	40 (83.3%)	0.2	8 (88.9%)	46 (76.7%)	0.4	7 (87.5%)	46 (78.0%)	0.5	3 (60.0%)	54 (77.1%)	0.4	5 (55.6%)	37 (77.1%)	0.2	3 (75.0%)	30 (73.2%)	0.9	3 (100.0%)	51 (78.5%)	0.4	12 (92.3%)	30 (71.4%)	0.06
Institution responsive to feedback?	2 (13.3%)	31 (64.6%)	0.001	4 (44.4%)	31 (51.7%)	0.7	4 (50.0%)	29 (49.2%)	0.96	2 (40.0%)	35 (50.0%)	0.7	4 (44.4%)	24 (50.0%)	0.8	2 (50.0%)	20 (48.8%)	0.96	2 (66.7%)	31 (47.7%)	0.5	6 (46.2%)	21 (50.0%)	0.8
Dept responsive to feedback?	6 (40.0%)	26 (54.2%)	0.3	1 (11.1%)	36 (60.0%)	0.006	1 (12.5%)	32 (54.2%)	0.03	2 (40.0%)	37 (52.9%)	0.6	4 (44.4%)	23 (47.9%)	0.8	1 (25.0%0	23 (56.1%)	0.2	1 (33.3%)	33 (50.8%)	0.6	4 (30.8%0	24 (57.1%)	0.1

## Discussion

The aim of this survey was to assess the experience, training, responsibilities, and contribution of neurosurgery residency program coordinators. To our knowledge, no such study has been carried out previously. The importance of such a survey is in the increasing role that coordinators play a role in managing neurosurgical staff, monitoring regulatory affairs for accreditation, maintaining budgets, and performing human resources tasks, all of which allow departments to function and directly impact patient care.

The response rate of 59% is consistent with surveys of program coordinators in other fields [1-3]. Overall, neurosurgery program coordinators are fairly experienced in their position, with >50% reporting working in their current position for ≥5 years. This experience is similar to program coordinators in other surgical subspecialties [2]. Additionally, neurosurgery coordinators normally manage one department, and the majority of coordinators manage less than three fellowship or residency programs.

Also consistent with other studies, residency program coordinators surveyed in our study reported inadequate training from both the institution and the department [1,3]. A barrier for training may result from the lack of a standardized definition of program coordinator responsibilities [9]. With different standards across specialties and some specialties only requiring that a coordinator simply be in place at a department, heterogeneity between coordinator roles will continue to exist [9]. Many coordinators, including those surveyed in our study, are responsible for managerial tasks not directly related to the residency/fellowships they manage [1-3]. As one coordinator highlighted in the response to our survey, handling departmental duties on top of managing the residency program felt like “extraneous” responsibilities. Coordinators reported that when these departmental responsibilities were relieved, they were much more effective in their role as residency program coordinator. Delineating work responsibilities at a departmental, institutional, and/or national level is warranted.

Workplace training and responsiveness to feedback were common threads that impacted the valuation of program coordinators. The lack of formal training, standardized qualifications and expectations, or standardized job descriptions leaves coordinators with responsibilities that take their focus away from residency program management, a demanding task in and of itself. Without formal training, coordinators can quickly become burned out from many tasks that they do not feel qualified to manage. Respondents to our survey with inadequate department or institutional training correlated with a greater likelihood of feeling undervalued by the institution, department, department chair, and program director(s). Coordinators were more likely to feel undervalued by the institution or department if either was less responsive to feedback. A longer career in the position predicted a greater likelihood of feeling valued by the program director(s), very likely correlating with lower coordinator turnover and a better working relationship between coordinators and directors. Interestingly, no specific factors could be identified to suggest coordinators were undervalued by other faculty, fellows, residents, or other staff. These findings suggest that mechanisms to improve the onsite training of coordinators, specific to their job descriptions, and direct interaction with institutional administrators and other program leaders would be important to increase perceived valuation. Likely, as seen with longer lengths of employment and greater valuation from program directors, improved self-valuation results in greater job retention, although this was not specifically queried in this survey.

Coordinators are also placed in a difficult and fast-paced educational environment, one that requires them to juggle working with residents and fellows, program directors, department chairs, and other faculty and staff. Our survey demonstrated that coordinators do not always feel valued by those with whom they work. Over 90% of coordinators completely or partially agreed to feeling valued by program directors with 84% reporting feeling valued by residents; however, the subjective perception of value decreased when coordinators were asked whether they agreed with feeling valued by the department chair, other faculty and staff, fellows, or the department and institution as a whole. In addition to this lack of perceived value, many departments in the field of neurosurgery do not offer a career advancement track for coordinators. This provides less incentive to receive certifications and additional training such as TAGME certification. Our data demonstrate that <15% of neurosurgery program coordinators are certified as such. If neurosurgery program coordinators are not properly valued and trained, the quality of residency program management may decrease at a time when residency program administration is becoming more complex.

The high turnover rate among program coordinators demonstrated in other studies may be related to the lack of respect that coordinators reported they receive from those within the department [1,3]. The complexity and heterogeneity of the role and lack of adequate training, coupled with a decreased perception of departmental value, places tremendous strain on a program coordinator. Half of the coordinators in this survey reported that their feedback to improve the position of program coordinator is often not regarded by the department or institution. This may lead to a perception that departments and institutions do not value the role of a program coordinator even if the ability to produce a policy change was limited. This could be addressed with a simple but formal mechanism to hear out coordinator questions and concerns.

The limitations of this study include a subjective method of assessing roles, responsibilities, and perceived departmental value. Not all coordinators responded to this survey, which may result in a selection bias with viewpoints shared by a vocal number of individuals rather than the total. Further studies should investigate the training of residency program coordinators as well as possible solutions for the increasing managerial tasks among coordinators, which distract from the time involved in managing residency and fellowship programs.

## Conclusions

Neurosurgical residency program coordinators play an important role with regards to the function of the residency program and department, which impacts patients. Coordinators reported a wide range of experience and responsibilities within their department, with the majority reporting no formal or inadequate training for their current position. A significant number also reported not feeling valued by members of their department and nearly half agreed that departments and institutions are not receptive to their feedback about how to improve the role of coordinators. These findings directly impact the work product of the coordinators and serve as areas for improvement. As coordinators continue to play a large role in the management and accreditation of their departments, further exploration into strategies to optimize their training and role is warranted.
